# Dysregulation of Niacin-Derived NAD^+^ Salvage Pathway Markers (CD38, NAMPT, SIRT1) Across Albuminuria Stages in Type 2 Diabetes

**DOI:** 10.3390/medicina61122089

**Published:** 2025-11-24

**Authors:** Bader Huwaimel, Saad Alqarni, Amr S. Abouzied, Ali Alghubayshi, Talal Alotaibi, Ahmed Elshafei, Marwa Yassien, Mohamed Nasr, Emad Gamil Khidr

**Affiliations:** 1 Department of Pharmaceutical Chemistry, College of Pharmacy, University of Ha’il, Hail 81442, Saudi Arabia; b.huwaimel@uoh.edu.sa (B.H.); s.alqarni@uoh.edu.sa (S.A.); as.ibrahim@uoh.edu.sa (A.S.A.); 2 Medical and Diagnostic Research Center, University of Ha’il, Hail 55473, Saudi Arabia; a.alghubyshi@uoh.edu.sa (A.A.); t.alotaibi@uoh.edu.sa (T.A.); 3 Department of Clinical Pharmacy, College of Pharmacy, University of Ha’il, Hail 81442, Saudi Arabia; 4 Department of Biochemistry and Molecular Biology, Faculty of Pharmacy for Boys, Al-Azhar University, Cairo 4434103, Egypt; ahmed.elshafei@azhar.edu.eg; 5 Department of Endocrinology and Metabolism, Faculty of Medicine for Girls, Al-Azhar University, Cairo 4434103, Egypt; marwayassien87@gmail.com; 6 Department of Histology, Faculty of Medicine, Al-Azhar University, Cairo 4434103, Egypt; mohamednasr2394.el@azhar.edu.eg

**Keywords:** type 2 diabetes, diabetic nephropathy, NAD^+^ salvage pathway, CD38, NAMPT, SIRT1, mitochondrial dysfunction

## Abstract

*Background and Objectives*: Diabetic nephropathy (DN) is a major cause of end-stage renal disease, yet its molecular basis remains unclear. Nicotinamide adenine dinucleotide (NAD^+^) metabolism is crucial for energy regulation, redox balance, and inflammation. This study investigated the dysregulation of key NAD^+^ salvage enzymes (CD38, NAMPT, and SIRT1) across albuminuria stages in type 2 diabetes (T2D). *Materials and Methods*: A cross-sectional study was conducted on 225 participants: healthy controls (n = 45), T2D with normoalbuminuria (n = 60), microalbuminuria (n = 60), and macroalbuminuria (n = 60). Serum CD38, NAMPT, and SIRT1 were measured by ELISA, while CD38 and SIRT1 gene expression in peripheral blood mononuclear cells was analyzed by qPCR. *Results*: CD38 and NAMPT levels increased progressively with albuminuria, whereas SIRT1 levels declined significantly. CD38 and NAMPT correlated positively with HbA1c, creatinine, and urinary albumin-to-creatinine ratio (UACR), while SIRT1 showed inverse correlations and a positive association with eGFR. Regression analysis identified CD38 and NAMPT as independent positive predictors of albuminuria, and SIRT1 as a negative predictor. ROC analysis revealed strong diagnostic performance for CD38 (AUC = 0.89) and SIRT1 (AUC = 0.88). *Conclusions*: These findings highlight disrupted NAD^+^ salvage pathways in DN and suggest that restoring NAD^+^ balance, through CD38 inhibition, SIRT1 activation, or NAD^+^ precursor supplementation, may offer promising renoprotective strategies.

## 1. Introduction

Diabetic nephropathy (DN), known as diabetic kidney disease, is a significant microvascular consequence of diabetes mellitus and a primary cause of end-stage renal disease globally, impacting around 20–40% of individuals [1,2]. The global burden of diabetes is paralleled by variable levels of awareness and prevention practices among different populations, including those in the Middle East [3]. There is a lot of geographic diversity in how common it is, with recent meta-analyses showing that the global rates are between 21% and 28% [4]. The clinical evolution of DN often involves a transition from normoalbuminuria to microalbuminuria, and eventually to macroalbuminuria, frequently staying asymptomatic until later stages. This highlights the pressing necessity for innovative biomarkers to facilitate prompt detection and risk categorization.

The pathophysiology of DN entails a complex interaction of metabolic abnormalities, oxidative stress, and chronic inflammation. There is more and more proof that nicotinamide adenine dinucleotide (NAD^+^) metabolism is a key player in how cells manage their energy and respond to stress in this situation [5,6]. In addition to its traditional role as an electron carrier in energy metabolism, NAD^+^ is also an essential cofactor for enzymes like sirtuins, poly (ADP-ribose) polymerases (PARPs), and CD38 (Cluster of Differentiation 38). These enzymes work together to affect aging, inflammation, and mitochondrial function [7,8].

In mammalian kidneys, the NAD^+^ salvage pathway is the most important. It recycles nicotinamide (NAM) into NAD^+^ with the help of nicotinamide phosphoribosyltransferase (NAMPT) and NMN adenylyltransferases (NMNATs) [9,10]. Disruption within this system, especially the imbalance between making and using NAD^+^, have been linked to both aging and diabetes [11]. CD38, NAMPT, and Sirtuin 1 (SIRT1) are some of the enzymes that are involved. They are important points of control and have been linked to more and more diabetic problems.

CD38, a major NAD^+^ hydrolase, contributes to NAD^+^ depletion and subsequent inflammatory activation. Its expression rises in both aging and diabetes, and experimental studies have shown that CD38 upregulation under hyperglycemic conditions reduces the NAD^+^/NADH ratio, promotes mitochondrial dysfunction, and exacerbates renal tubular injury [12,13]. Conversely, pharmacological inhibition of CD38 restores NAD^+^ levels and confers renoprotection.

NAMPT serves as the rate-limiting enzyme of the salvage pathway, with dual roles in DN. While NAMPT upregulation can act as a compensatory mechanism to preserve NAD^+^ pools during metabolic stress, excessive activity has been associated with inflammation and fibrosis [14]. Thus, the balance between NAMPT-mediated NAD^+^ production and CD38-mediated consumption is critical in determining renal outcomes.

SIRT1, a NAD^+^-dependent deacetylase, serves as a crucial modulator of kidney protection. It controls podocyte integrity, tubular cell survival, and inflammatory signaling [15,16]. Notably, SIRT1 downregulation in proximal tubules occurs early in diabetes, precedes albuminuria, and contributes to glomerular dysfunction via altered tubulo-glomerular crosstalk [17].

Even with these findings, the connection between the severity of albuminuria and indicators of the NAD^+^ salvage pathway is still not well understood. Most current research is confined to animal models or small patient populations, hence limiting their translational applicability [18]. Albuminuria is a clinical indicator of DN development and a proxy for cellular dysfunction. Evaluating NAD^+^ pathway markers at various phases of albuminuria may provide significant diagnostic and therapeutic insights.

**Figure 1:**
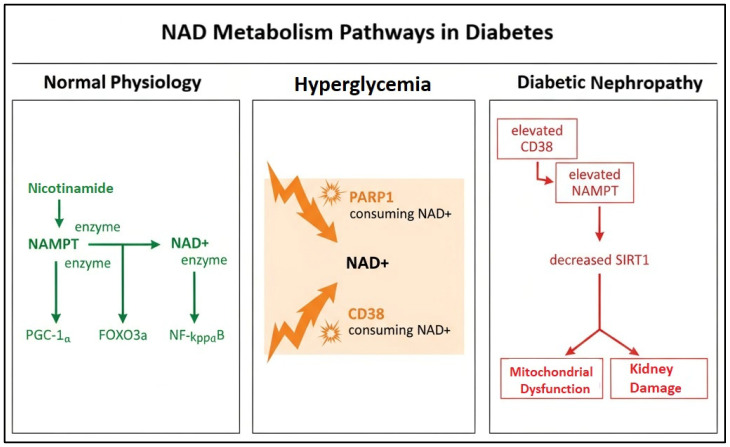
Schematic illustration of NAD^+^ metabolism pathways in diabetes.

An overview of the proposed NAD^+^ metabolic alterations in diabetes is illustrated in Figure 1.

This study seeks to systematically examine blood concentrations and gene expression of CD38, NAMPT, and SIRT1 across several stages of albuminuria in individuals with type 2 diabetes mellitus. This study aims to clarify the molecular modifications of the NAD^+^ salvage pathway in diabetic nephropathy progression by combining protein quantification using ELISA and gene expression profiling via qPCR, while also investigating their potential as innovative biomarkers and therapeutic targets.

The figure shows the transition from normal physiology to hyperglycemia and diabetic nephropathy, highlighting increased CD38- and PARP1-mediated NAD^+^ consumption, compensatory NAMPT upregulation, and subsequent SIRT1 depletion leading to mitochondrial dysfunction and renal injury.

## 2. Subjects and Methods

### 2.1. Study Design and Population

This case–control, cross-sectional study was conducted on a total of 225 participants recruited from Endocrinology and Metabolism Department at Al-Zahraa Hospital, Al-Azhar University, Cairo, Egypt. Participants were divided into four groups according to diabetes status and urinary albumin-to-creatinine ratio (UACR): Healthy non-diabetic controls (n = 45), patients with type 2 diabetes (T2D) and normoalbuminuria (UACR < 30 mg/g; n = 60), T2D with microalbuminuria (UACR 30–300 mg/g; n = 60), and T2D with macroalbuminuria (UACR > 300 mg/g; n = 60).

The diagnosis of T2D was based on the American Diabetes Association 2024 criteria [19]. Albuminuria classification was performed according to KDIGO 2024 guidelines [20].

Inclusion criteria included age between 40 and 65 years with an established diagnosis of T2D for at least five years, while exclusion criteria comprised type 1 diabetes mellitus, a history of chronic kidney disease unrelated to diabetes such as glomerulonephritis or polycystic kidney disease, advanced liver disease, autoimmune disorders, malignancy, acute infection, or the current use of NAD^+^ precursors, antioxidants, or immunomodulatory drugs.

### 2.2. Clinical and Biochemical Assessment

Demographic and clinical data (age, sex, BMI, duration of diabetes, blood pressure) were recorded. Blood samples were collected after overnight fasting. Biochemical parameters including fasting glucose, HbA1c, serum creatinine, and lipid profile were measured using standard automated methods. Estimated glomerular filtration rate (eGFR) was calculated using the CKD-EPI equation [21]. Urinary albumin and creatinine were determined from spot urine samples to calculate UACR.

Serum levels of CD38, NAMPT, and SIRT1 were quantitatively measured using commercially available enzyme-linked immunosorbent assay (ELISA) kits according to the manufacturer’s instructions, including the Human CD38 ELISA Kit (Abcam, Cambridge, UK; ab272389), the Human Visfatin/NAMPT ELISA Kit (Antibodies-online, Aachen, Germany; ABIN6574184), and the Human SIRT1 ELISA Kit (Abcam, Cambridge, UK; ab171573).

### 2.3. Gene Expression Analysis

Peripheral blood mononuclear cells (PBMCs) were isolated from whole blood samples by density gradient centrifugation using Ficoll-Paque PLUS (GE Healthcare, Uppsala, Sweden). The isolated PBMCs were washed twice with phosphate-buffered saline (PBS) and immediately processed for RNA extraction. Total RNA was extracted using the miRNeasy Mini Kit (Qiagen, Hilden, Germany) according to the manufacturer’s protocol. Briefly, cells were lysed with QIAzol reagent, followed by phase separation with chloroform. The aqueous phase was mixed with ethanol and loaded onto silica spin columns. RNA was eluted in RNase-free water and quantified using a NanoDrop spectrophotometer (Thermo Fisher Scientific, Waltham, MA, USA). The purity of RNA was confirmed by A260/280 and A260/230 ratios, with acceptable values between 1.8–2.1 and >1.7, respectively.

Complementary DNA (cDNA) was synthesized from 1 µg of total RNA using the miScript II RT Kit (Qiagen, Hilden, Germany) following the manufacturer’s instructions. The reaction was incubated at 37 °C for 60 min, followed by enzyme inactivation at 95 °C for 5 min. The synthesized cDNA was stored at −80 °C until further use.

Quantitative real-time PCR was performed using the miScript SYBR Green PCR Kit (Qiagen, Hilden, Germany) and gene-specific primers for CD38, NAMPT, and SIRT1, with GAPDH as the housekeeping gene. Amplification was carried out in a Rotor-Gene Q thermocycler (Qiagen, Hilden, Germany) under the following cycling conditions: initial activation at 95 °C for 15 min, followed by 40 cycles of denaturation at 94 °C for 15 s, annealing at 55 °C for 30 s, and extension at 70 °C for 30 s. Melt curve analysis was included to verify amplification specificity.

Relative gene expression was analyzed using the ΔΔCt method, and fold changes were expressed as relative quantification values with the healthy control group serving as reference.

The study was conducted following the principles outlined in the Declaration of Helsinki. Approval was obtained from the Ethics Committee of Faculty of Medicine, Al-Azhar University, Egypt and written informed consent was secured from all participants prior to enrolment.

### 2.4. Statistical Analysis

The data were analyzed using GraphPad Prism software, version 10.4 (San Diego, CA, USA) and The Statistic Package for the Social Sciences (SPSS) software, version 24 (Armonk, NY, USA). Results were expressed as mean ± standard deviation (SD) or mean (percentage). The normality of data distribution across groups and subgroups was assessed using the D’Agostino and Pearson omnibus normality test. While the overall data for each RNA passed the normality test, variability was observed in subgroup distributions. Group comparisons were performed using one-way ANOVA with Tukey’s post-hoc test or Kruskal–Wallis with Dunn’s post-hoc test. Correlations between NAD^+^ markers and clinical parameters (UACR, HbA1c, eGFR) were assessed using Pearson’s or Spearman’s correlation analysis. Receiver operating characteristic (ROC) curves were generated to evaluate the diagnostic performance of NAD^+^ markers in distinguishing albuminuria stages. Multiple linear regression analysis was performed to identify independent predictors of albuminuria. To control for potential Type I error inflation due to multiple comparisons, *p*-values from group and correlation analyses were adjusted using the Benjamini–Hochberg false discovery rate (FDR) procedure, with q < 0.05 considered significant. Multiple regression models were further adjusted for age, BMI, and diabetes duration to control for potential confounding effects. The optimal cut-off points in ROC curve analyses were determined using the Youden index to maximize diagnostic accuracy. The sample size (n = 225) was considered sufficient to ensure a statistical power greater than 0.8 at α = 0.05. A *p*-value < 0.05 was considered statistically significant.

## 3. Results

Baseline demographic and clinical characteristics of the study groups are summarized in Table 1. There were no significant differences in age or sex distribution among the groups. BMI showed a significant stepwise increase from controls to macroalbuminuria patients. Fasting blood glucose and HbA1c were significantly elevated in all diabetic groups compared with controls, with the highest levels observed in the macroalbuminuria group. Similarly, serum urea and creatinine demonstrated significant increments across albuminuria stages, accompanied by a marked decline in estimated eGFR.

As presented in Table 2, serum CD38 and NAMPT levels were significantly upregulated across albuminuria stages, whereas SIRT1 levels showed a consistent decline (*p* < 0.001 for all comparisons). CD38 increased nearly eightfold from controls (74.2 ± 11.7 ng/mL) to macroalbuminuria (615 ± 46.6 ng/mL), while NAMPT levels rose more than fivefold (7.53 ± 1.71 ng/mL to 43.4 ± 3.95 ng/mL). In contrast, SIRT1 was markedly reduced, reaching 0.65 ± 0.20 ng/mL in macroalbuminuria compared to 3.52 ± 0.87 ng/mL in controls.

Relative gene expression levels are shown in Table 3. CD38 mRNA expression increased progressively with albuminuria severity, from 2.4-fold in normoalbuminuria to 8.5-fold in macroalbuminuria compared with controls (*p* < 0.001). Conversely, SIRT1 expression was significantly downregulated, decreasing from 0.6-fold in normoalbuminuria to 0.22-fold in macroalbuminuria (*p* < 0.001). These findings mirror the protein-level changes observed in serum.

Correlation coefficients between NAD^+^ salvage markers and clinical parameters are detailed in Table 4. CD38 and NAMPT showed strong positive correlations with HbA1c, serum urea, creatinine, and UACR, and negative correlations with eGFR. In contrast, SIRT1 exhibited inverse correlations with HbA1c, renal function markers, and UACR, while correlating positively with eGFR.

Multiple linear regression analysis (Table 5) identified CD38 and NAMPT as independent positive predictors of UACR, while SIRT1 emerged as a negative predictor. Traditional parameters including HbA1c, serum urea, creatinine, and eGFR also retained significance. After adjusting for age, BMI, and diabetes duration in the multivariable model, CD38, NAMPT, and SIRT1 remained strong independent predictors of albuminuria, confirming that their associations were not confounded by these clinical covariates.

**Figure 2:**
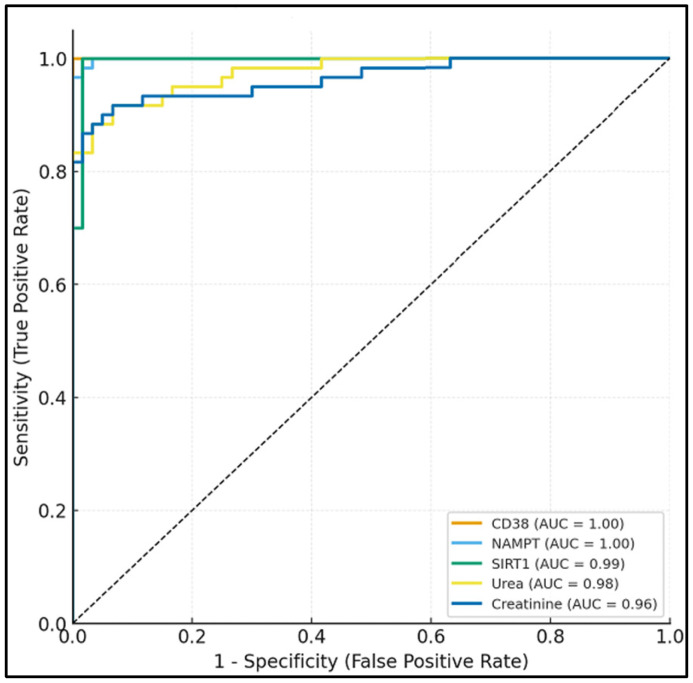
Receiver operating characteristic (ROC) curves showing diagnostic performance of serum NAD^+^ pathway markers (CD38, NAMPT, SIRT1) versus conventional renal markers for diabetic nephropathy discrimination.

Receiver operating characteristic (ROC) analysis (Table 6, Figure 2) demonstrated that CD38 (AUC = 0.89, 95% CI: 0.83–0.94) and SIRT1 (AUC = 0.88, 95% CI: 0.82–0.93) showed the best discriminatory ability for DN, with sensitivities of 85% and 83%, respectively, and specificities of 82% and 80%. NAMPT also demonstrated good diagnostic performance (AUC = 0.84, 95% CI: 0.77–0.90). In comparison, conventional markers such as serum urea (AUC = 0.76) and creatinine (AUC = 0.78) exhibited lower diagnostic accuracy.

## 4. Discussion

The present study revealed a distinctive dysregulation of the NAD^+^ salvage pathway across albuminuria stages in T2D, characterized by a stepwise increase in circulating and transcript levels of CD38 and NAMPT together with a marked decline in SIRT1. These coordinated alterations indicate a metabolic inflammatory imbalance that underpins DN progression and reflect the cellular attempt to maintain redox and energy homeostasis under chronic hyperglycemia, consistent with previous reports emphasizing the role of novel circulating biomarkers in predicting DN progression [22].

Hyperglycemia-induced oxidative stress is known to activate NAD^+^-consuming enzymes, particularly CD38. In accordance with our observation of an approximately eight-fold increase in serum CD38, other studies have shown that CD38 expression is significantly elevated in glomerular and tubular compartments, which accelerates NAD^+^ breakdown and induces mitochondrial dysfunction and inflammatory cascades [12,23]. Consistent with these data, our findings showed strong positive correlations between CD38 and indices of renal injury (UACR, serum creatinine) and inverse correlations with eGFR, supporting the notion that CD38 upregulation mechanistically links oxidative stress to renal damage through NAD^+^ depletion and impaired mitochondrial redox cycling.

Parallel to CD38 activation, we observed a pronounced reduction in SIRT1 at both mRNA and protein levels across albuminuria stages. SIRT1 is a NAD^+^-dependent deacetylase that preserves mitochondrial biogenesis, antioxidant defenses, and podocyte integrity. When SIRT1 activity is reduced, mitochondrial renewal led by PGC-1α stops, and FOXO1 and NF-κB become more acetylated, which leads to apoptosis and inflammation [24]. The inverse correlation between SIRT1 and albuminuria reported in this study reflects findings from human and experimental research that indicate an early drop in tubular SIRT1 prior to the onset of nephropathy, underscoring its essential function in preserving renal metabolic health. The inverse regulation of SIRT1 and CD38 underscores a dysfunctional NAD^+^-SIRT1 signaling disruption that undermines mitochondrial resilience in diabetic nephropathy.

The increase in NAMPT in our diabetic groups provides additional evidence for the dynamic remodeling of NAD^+^ metabolism. NAMPT is the rate-limiting enzyme of the salvage pathway. It rises at first to make up for the loss of NAD^+^, but when it is overexpressed for a long time, it stimulates NF-κB p65 signaling and causes genes that cause inflammation and fibrosis to be expressed [14]. The positive correlation between NAMPT and UACR suggests that this adaptive response becomes maladaptive, contributing to extracellular-matrix accumulation and glomerulosclerosis. Together, the imbalance between NAMPT-mediated biosynthesis and CD38-driven consumption, compounded by diminished SIRT1 utilization, establishes a state of persistent energetic stress in renal tissue. This metabolic disruption does not occur in isolation, as diabetic nephropathy often coexists with other comorbidities such as hypertension, cardiovascular disease, and dyslipidemia, which further accelerate renal deterioration [25].

Beyond these enzymatic interactions, oxidative DNA damage activates PARP1, another potent NAD^+^-consuming enzyme, further depleting NAD^+^ pools and worsening energy failure [26]. Recent work by Fan et al. (2024) further supports this link, demonstrating that NAD^+^ depletion drives mitochondrial fragmentation and defective autophagy in diabetic kidneys through PARP1 overactivation [27]. Our results therefore integrate these molecular events into a coherent model whereby chronic hyperglycemia provokes NAD^+^ depletion, mitochondrial dysfunction, and fibrosis through converging CD38-NAMPT-SIRT1 perturbations.

In diagnostic terms, both CD38 and SIRT1 exhibited higher discriminatory power than conventional renal markers such as serum urea or creatinine, underscoring their potential as early, sensitive biomarkers for DN staging. Integrating these markers into screening algorithms could improve early detection and therapeutic monitoring. Future multicenter cohorts are warranted to validate cut-off values, standardize measurement protocols, and assess whether integrating these biomarkers improves risk prediction beyond conventional renal indices.

From a translational perspective, restoration of NAD^+^ homeostasis represents a promising therapeutic avenue in diabetic nephropathy. CD38 inhibition has gained attention as a means of preserving intracellular NAD^+^ levels and mitigating renal inflammation. Natural compounds such as apigenin and small-molecule inhibitors like 78c have shown efficacy in preclinical models by suppressing NAD^+^ hydrolysis and improving renal mitochondrial function [28,29]. Moreover, the monoclonal anti-CD38 antibody daratumumab, currently approved for multiple myeloma, has demonstrated renal function improvement in patients with myeloma-related kidney injury, although its application in diabetic nephropathy remains experimental due to potential immune effects [30,31].

Another promising strategy involves NAD^+^ precursor supplementation, particularly nicotinamide riboside (NR) and nicotinamide mononucleotide (NMN), both of which have shown favorable safety profiles in Phase I–II clinical trials. These precursors effectively elevate circulating NAD^+^ levels, improve insulin sensitivity, and enhance mitochondrial function [29,32]. Notably, Yasuda et al. (2021) demonstrated that short-term NMN administration in diabetic mice induced a long-lasting “legacy effect” through epigenetic reprogramming, resulting in sustained renoprotection despite persistent hyperglycemia [33].

Additionally, SIRT1 activators such as resveratrol, and newer analogues like SRT1720 and SRT2104, have been shown to attenuate renal oxidative stress and fibrosis in diabetic models, though clinical translation remains limited by pharmacokinetic challenges [34]. Moreover, recent evidence suggests that SGLT2 inhibitors, now a mainstay therapy for diabetic nephropathy, may exert part of their renoprotective effects through restoration of renal SIRT1 activity and improvement of mitochondrial integrity, thereby indirectly modulating NAD^+^ metabolism [35,36]. This potential overlap between glucose transport inhibition and NAD^+^-SIRT1 pathway activation further supports the therapeutic relevance of our findings.

Certain limitations merit acknowledgment. The cross-sectional design restricts causal inference between NAD^+^-marker alterations and disease progression. While we measured CD38 and SIRT1 gene expression in PBMCs, this study did not include NAMPT gene expression analysis, which limits the ability to directly compare mRNA and protein levels across all NAD^+^ salvage pathway markers. Future studies incorporating NAMPT transcript quantification will be valuable to clarify post-transcriptional regulation and strengthen mechanistic interpretation. Most importantly, we did not directly quantify NAD^+^ or NADH levels in serum or PBMCs, which would have provided direct biochemical confirmation of cellular NAD^+^ depletion. Instead, NAD^+^ dysregulation was inferred from alterations in the key enzymatic markers (CD38, NAMPT, and SIRT1), consistent with previously established mechanistic evidence. While this indirect approach is informative and commonly used in clinical translational studies, direct measurement of the NAD^+^/NADH ratio using colorimetric enzymatic assays or LC–MS would further strengthen mechanistic interpretation. Future studies should incorporate direct NAD^+^ metabolomics in both circulation and kidney tissue alongside enzyme profiling to better elucidate the causal relationship between enzymatic dysregulation and tissue NAD^+^ depletion. Potential uncontrolled confounders such as comorbidities, and genetic polymorphisms may have influenced biomarker levels. Single-center recruitment limits generalizability, and functional NAD^+^ quantification or PARP-activity assays were not performed, which would further substantiate mechanistic interpretation. Kidney biopsy samples were not available, precluding tissue-level validation of the observed circulating alterations. Medication data, including SGLT2 inhibitor and GLP-1 receptor agonist use, were not uniformly available across all participants and therefore could not be included as covariates in multivariable models, which may have introduced residual confounding. Future multi-center studies integrating renal tissue analysis, longitudinal biomarker tracking, and interventional trials with NAD^+^ precursors or CD38 inhibitors are warranted to validate these findings. Despite these constraints, the congruence between our biochemical and molecular findings with prior mechanistic evidence supports the robustness of the observed relationships.

## 5. Conclusions

Taken together, the present findings provide integrative human evidence that coordinated upregulation of CD38 and NAMPT together with depletion of SIRT1 constitutes a molecular signature of NAD^+^-salvage-pathway disruption that mirrors the severity of albuminuria and renal dysfunction in type 2 diabetes. This triad encapsulates the metabolic and inflammatory underpinnings of diabetic nephropathy and highlights a tractable target for biomarker-guided and NAD^+^-restoration therapies. Longitudinal studies incorporating functional NAD^+^ metrics and interventional modulation are warranted to confirm causality and therapeutic potential.

## Figures and Tables

**Table 1:** Demographic, Clinical, and Biochemical Profiles of the Study Groups.

Variables	Controls (n = 45)	T2D Normoalbuminuria (n = 60)	T2D Microalbuminuria (n = 60)	T2D Macroalbuminuria (n = 60)	*p*-Value
Sex					
Male	23 (51.1%)	29 (48.3%)	32 (53.3%)	33 (55%)	0.897
Female	22 (48.9%)	31 (51.7%)	28 (46.7%)	27 (45%)	
Age (years)	48.10 ± 5.78	47.9 ± 5.50	48.90 ± 4.72	49.90 ± 4.72	0.115
BMI (kg/m^2^)	24.28 ± 0.51	24.65 ± 0.50	25.80 ± 0.47	27.20 ± 0.29	<0.001 *
Diabetes duration (years)	–	4.35 ± 1.26	11.14 ± 2.94 ^b^	18.17 ± 3.42 ^b,c^	<0.001 *
FBG (mg/dL)	90.24 ± 5.94	185 ± 22.78 ^a^	255 ± 43.87 ^a,b^	279.50 ± 34.13 ^a,b,c^	<0.001 *
HbA1c (%)	5.27 ± 0.19	6.9 ± 0.28 ^a^	7.19 ± 0.34 ^a,b^	8.86 ± 0.39 ^a,b,c^	<0.001 *
Serum urea (mg/dL)	24.70 ± 2.94	27.80 ± 2.15 ^a^	44.90 ± 3.50 ^a,b^	65.20 ± 4.28 ^a,b,c^	<0.001 *
Serum creatinine (mg/dL)	0.89 ± 0.12	1.04 ± 0.14 ^a^	1.38 ± 0.19 ^a,b^	2.09 ± 0.18 ^a,b,c^	<0.001 *
UACR (mg/g)	14.23 ± 1.84	22.80 ± 3.05 ^a^	114.10 ± 38 ^a,b^	356.3 ± 19.93 ^a,b,c^	<0.001 *
eGFR (mL/min/1.73 m^2^)	93.30 ± 15.40	48.70 ± 17.20 ^a^	53.80 ± 11.60 ^a,b^	32 ± 6.18 ^a,b,c^	<0.001 *

T2D: type 2 diabetes; BMI: body mass index; FBG: fasting blood glucose; HbA1c: glycated hemoglobin; UACR: urinary albumin to creatinine ratio; eGFR: estimated glomerular filtration rate. *p*-value represents overall significance among the three groups (one-way ANOVA or Kruskal–Wallis). * indicates statistical significance at *p* < 0.05. Different superscript letters (a, b, c) indicate significant pairwise differences between specific groups based on post hoc Tukey’s or Dunn’s test (*p* < 0.05): a = vs. controls, b = vs. T2D with normoalbuminuria, c = vs. T2D with microalbuminuria.

**Table 2:** Serum levels of NAD^+^ salvage pathway markers across study groups.

Markers	Controls (n = 45)	T2D Normoalbuminuria (n = 60)	T2D Microalbuminuria (n = 60)	T2D Macroalbuminuria (n = 60)	*p*-Value
CD38 (ng/mL)	74.20 ± 11.70	202 ± 14.20 ^a^	385 ± 30.10 ^a,b^	615 ± 46.60 ^a,b,c^	<0.001 *
NAMPT (ng/mL)	7.53 ± 1.71	15.10 ± 1.59 ^a^	25.90 ± 2.55 ^a,b^	43.40 ± 3.95 ^a,b,c^	<0.001 *
SIRT1 (ng/mL)	3.52 ± 0.87	1.84 ± 0.49 ^a^	1.28 ± 0.31 ^a,b^	0.65 ± 0.20 ^a,b,c^	<0.001 *

T2D: type 2 diabetes; CD38: Cluster of Differentiation 38; NAMPT: Nicotinamide phosphoribosyl transferase; SIRT1: Sirtuin 1. *p*-value represents overall significance among the three groups (one-way ANOVA or Kruskal–Wallis). * indicates statistical significance at *p* < 0.05. Different superscript letters (a, b, c) indicate significant pairwise differences between specific groups based on post hoc Tukey’s or Dunn’s test (*p* < 0.05): a = vs. controls, b = vs. T2D with normoalbuminuria, c = vs. T2D with microalbuminuria.

**Table 3:** Relative gene expression (fold change) of CD38 and SIRT1 across study groups.

Markers	Controls (n = 45)	T2D Normoalbuminuria (n = 60)	T2D Microalbuminuria (n = 60)	T2D Macroalbuminuria (n = 60)	*p*-Value
CD38	0.97	2.40 ± 0.50 ^a^	5.02 ± 1.30 ^a,b^	8.50 ± 1.50 ^a,b,c^	<0.001 *
SIRT1	1.02	0.60 ± 0.11 ^a^	0.38 ± 0.14 ^a,b^	0.22 ± 0.12 ^a,b,c^	<0.001 *

T2D: Type 2 Diabetes; CD38: Cluster of Differentiation 38; SIRT1: Sirtuin 1. Controls served as reference group (fold change ≈ 1). *p*-value represents overall significance among the three groups (Kruskal–Wallis). * indicates statistical significance at *p* < 0.05. Different superscript letters (a, b, c) indicate significant pairwise differences between specific groups based on post hoc Dunn’s test (*p* < 0.05): a = vs. controls, b = vs. T2D with normoalbuminuria, c = vs. T2D with microalbuminuria.

**Table 4:** Correlation of NAD^+^ salvage pathway markers (CD38, NAMPT, and SIRT1) with clinical and biochemical parameters in T2D patient.

Parameter	CD38		NAMPT		SIRT1	
	R	*p*-Value	R	*p*-Value	R	*p*-Value
HbA1c (%)	0.50	<0.001	0.45	0.002	−0.48	0.001 *
Serum urea (mg/dL)	0.48	0.004	0.44	0.008	−0.46	0.002 *
Serum creatinine (mg/dL)	0.52	0.006	0.47	0.01	−0.53	0.003 *
UACR (mg/g)	0.65	<0.001	0.58	<0.001	−0.60	<0.001 *
eGFR (mL/min/1.73 m^2^)	−0.55	<0.001	−0.50	<0.001	0.52	<0.001 *

CD38: Cluster of Differentiation 38, NAMPT: Nicotinamide phosphoribosyl transferase, SIRT1: Sirtuin 1, HbA1c: glycated hemoglobin, UACR: urinary albumin to creatinine ratio, eGFR: estimated glomerular filtration rate. *: Statistically significant at *p*-value < 0.05.

**Table 5:** Multiple linear regression analysis of predictors of albuminuria (UACR) in T2D patients.

Variable	β (Standardized Coefficient)	SE	T	*p*-Value
Age (years)	0.08	0.05	1.52	0.131
BMI (kg/m^2^)	0.12	0.06	2.05	0.042 *
Diabetes duration (years)	0.16	0.05	3.15	0.002 *
HbA1c (%)	0.22	0.07	3.10	0.003 *
Serum urea (mg/dL)	0.15	0.06	2.45	0.015 *
Serum creatinine (mg/dL)	0.18	0.08	2.20	0.028 *
eGFR (mL/min/1.73 m^2^)	−0.20	0.06	−3.35	0.001 *
CD38 (ng/mL)	0.35	0.09	4.10	<0.001 *
NAMPT (ng/mL)	0.28	0.08	3.60	<0.001 *
SIRT1 (ng/mL)	−0.30	0.10	−3.00	0.004 *

BMI: body mass index; HbA1c: glycated hemoglobin; eGFR: estimated glomerular filtration rate; CD38: Cluster of Differentiation 38; NAMPT: Nicotinamide phosphoribosyl transferase; SIRT1: Sirtuin 1. β = standardized regression coefficient; SE = standard error; T = *t*-statistic. Model fit: R^2^ = 0.68, Adjusted R^2^ = 0.66, F = 38.2, *p* < 0.001. The model included age, BMI, and diabetes duration as additional covariates alongside conventional renal parameters. CD38, NAMPT, and SIRT1 remained independent predictors of albuminuria after full adjustment. *: Statistically significant at *p*-value < 0.05.

**Table 6:** Receiver operating characteristic (ROC) analysis of serum biomarkers and renal function parameters for discrimination of diabetic nephropathy.

Marker	AUC (95% CI)	Cut-Off	Sensitivity (%)	Specificity (%)	*p*-Value
CD38 (ng/mL)	0.89 (0.83–0.94)	>4.20	85	82	<0.001 *
NAMPT (ng/mL)	0.84 (0.77–0.90)	>2.10	80	78	<0.001 *
SIRT1 (ng/mL)	0.88 (0.82–0.93)	<0.45	83	80	<0.001 *
Serum Urea (mg/dL)	0.76 (0.68–0.83)	>42	70	72	0.004 *
Serum Creatinine (mg/dL)	0.78 (0.70–0.85)	>1.20	73	74	0.003 *

AUC: Area under the curve, CI: Confidence interval, CD38: Cluster of Differentiation 38, NAMPT: Nicotinamide phosphoribosyl transferase, SIRT1: Sirtuin 1. *: Statistically significant at *p*-value <0.05.

## Data Availability

The data that support the findings of this study are available on request from the corresponding author.

## Abbreviations

The following abbreviations are used in this manuscript:

DN	Diabetic nephropathy
CD38	Cluster of Differentiation 38
NAMPT	Nicotinamide phosphoribosyl transferase
SIRT1	Sirtuin 1
PARPs	Poly (ADP-ribose) polymerases
UACR	Urinary albumin-to-creatinine ratio
ROC	Receiver operating characteristic
AUC	Area under the curve
